# Severe Food Protein-Induced Enterocolitis Syndrome to Cow’s Milk in Infants

**DOI:** 10.3390/nu8010001

**Published:** 2015-12-22

**Authors:** Min Yang, Lanlan Geng, Zhaohui Xu, Peiyu Chen, Craig A. Friesen, Sitang Gong, Ding-You Li

**Affiliations:** 1 Department of Gastroenterology, Guangzhou Women and Children’s Medical Center, Guangzhou Medical University, Guangzhou 510623, China; ymlyxw@hotmail.com (M.Y.); genglan_2001@hotmail.com (L.G.); xuer_hui@126.com (Z.X.); chenpei.y@163.com (P.C.); 2 Division of Gastroenterology, Children’s Mercy Hospital, University of Missouri Kansas City School of Medicine, Kansas City, MO 64108, USA; cfriesen@cmh.edu

**Keywords:** cow’s milk protein allergy, food-protein-induced enterocolitis syndrome, infants

## Abstract

Cow’s milk is the most common cause of food-protein-induced enterocolitis syndrome (FPIES). The aim of this study was to examine the clinical features and treatment outcomes of infants with severe FPIES to cow’s milk. We reviewed all infants ≤12 months of age who were hospitalized and diagnosed with severe FPIES to cow’s milk between 1 January 2011 and 31 August 2014 in a tertiary Children’s Medical Center in China. Patients’ clinical features, feeding patterns, laboratory tests, and treatment outcomes were reviewed. A total of 12 infants met the inclusion criteria. All infants presented with diarrhea, edema, and hypoalbuminemia. Other main clinical manifestations included regurgitation/vomiting, skin rashes, low-grade fever, bloody and/or mucous stools, abdominal distention, and failure to thrive. They had clinical remission with resolution of diarrhea and significant increase of serum albumin after elimination of cow’s milk protein (CMP) from the diet. The majority of infants developed tolerance to the CMP challenge test after 12 months of avoidance. In conclusion, we reported the clinical experience of 12 infants with severe FPIES to cow’s milk, which resulted in malnutrition, hypoproteinemia, and failure to thrive. Prompt treatment with CMP-free formula is effective and leads to clinical remission of FPIES in infants.

## 1. Introduction

Cow’s milk protein allergy (CMPA) is the most common food allergy in infants and young children and can induce a diverse range of symptoms of variable intensity in infants, involving many different organ systems, mostly the gastrointestinal tract [1,2]. It is also the most common cause of food-protein-induced enterocolitis syndrome (FPIES) in infants, who usually present with recurrent vomiting, lethargy, pallor, diarrhea with blood and/or mucus, and dehydration with metabolic acidosis in the acute setting, and hypoalbuminemia and failure to thrive in a chronic form [3,4,5,6,7]. The diagnosis of FPIES to cow’s milk is made on the basis of clinical presentation and cow’s milk protein (CMP) avoidance/challenge; however, misdiagnosis and delayed diagnosis are common. Here, we reported the first clinical experience of Chinese infants with severe FPIES to cow’s milk.

## 2. Patients and Methods

The institutional ethics committee of Guangzhou Women and Children’s Medical Center approved this study protocol.

A retrospective case-series analysis was carried out in a tertiary children’s hospital in Guangzhou, China. We included all infants ≤12 months of age who were hospitalized and diagnosed with severe FPIES to cow’s milk between 1 January 2011 and 31 August 2014. Patients’ clinical features, feeding patterns, laboratory tests, and treatment outcomes were reviewed. All infants underwent routine evaluation for acute infection including blood culture for bacteria and fungus, stool culture for bacteria (*Escherichia coli*, Salmonella, Shigella, *Campylobacter* and Enterobacter aerogenes), and stool viral antigen tests (rotavirus and adenovirus). Patients with documented infection, sepsis, immunodeficiency, kidney, liver, and heart disorders were excluded.

The diagnosis of severe FPIES to cow’s milk was based on the criteria described by others: (a) repeated exposure to cow’s milk elicited repetitive vomiting and/or diarrhea within 24 h, without any other cause for the symptoms; (b) removal of CMP from the diet resulted in resolution of symptoms and a food challenge elicited vomiting and/or diarrhea within two weeks after administration of the food containing CMP to observe for any immediate and delayed clinical reactions [1,5,6,8,9]; (c) Infants had severe shock-like reactions with dehydration and metabolic acidosis in the acute setting and/or hypoalbuminemia and failure to thrive in a chronic form [1,7].

## 3. Results

As shown in Table 1, a total of 12 infants were diagnosed with severe FPIES to cow’s milk; the mean age of infants was 3.2 months, ranging from 29 days to 8 months, with male predominance. The majority (83.3%) of infants presented at <6 months of age. All infants presented with diarrhea from 5 to 20 times a day. Other common presenting symptoms included regurgitation/vomiting, low-grade fever, bloody and/or mucusy stools, and irritability. Physical examination showed skin rashes, abdominal distention/bloating, dehydration, and failure to thrive. All infants had edema and 7 (58.3%) had ascites assessed by abdominal ultrasound. Except for one infant on exclusive breast feeding, all others were on cow’s milk-based formula or mixed feeding. Laboratory tests showed that all infants (100%) had hypoproteinemia (25.3 to 45.5 g/L, normal reference 60 to 80 g/L), hypoalbuminemia (14.5 to 24.8 g/L, normal reference 35 to 50 g/L); 9 (75%) infants had decreased serum globulin (10.8 to 19.5 g/L, normal reference 20 to 29 g/L). Most patients (58.3%) had elevated WBC (≥12.5 × 10^9^/L, range 13.1 to 32.9 × 10^9^/L) and platelets (≥440 × 10^9^/L); 4 patients (33.3%) had anemia (HB < 90 g/L). Five patients (41.7%) had metabolic acidosis (HCO_3_^−^ <18 mmol/L, normal reference 21.3 to 24.8 mmol/L). Half of the infants had elevated total serum IgE but only 25% of them had positive serum allergen-specific IgE (sIgE) to cow’s milk protein. The course of disease before final diagnosis was from 10 days to 3 months. Five patients (41.7%) had misdiagnosis (two with necrotizing enterocolitis; one each for chronic diarrhea, acrodermatitis enteropathica, and chronic partial intestinal obstruction) and delayed diagnosis before an evaluation by a pediatric gastroenterologist.

Five (41.7%) were treated with amino acid-based formula, and another seven patients (58.3%) were treated with extensively hydrolyzed formula initially, but one transitioned to amino acid-based formula after two weeks because of poor response. All cow’s milk-based products were avoided. Eight patients (66.7%) were fed initially via nasogastric tube and then transitioned to oral feeding, whereas four patients (33.3%) were fed orally. During hospitalization, 10 infants (83.3%) received albumin infusion, with an average dose of 3.10 ± 2.31 g/kg. All infants had clinical remission with resolution of diarrhea. There were significant increases of serum total protein and albumin levels to 54.9 ± 10 and 36.7 ± 8.8 g/L (both *p* < 0.01), respectively, compared to pre-treatment baseline; six patients had serum globulin level improved, and three of them returned to the normal range. The average length of hospital stay was 17.8 ± 11.1 days (range 4 to 37 days).

At clinic follow-up from three months to two years, all infants underwent an open oral challenge test to CMP. The challenge procedure was performed with lactose-free CMP-containing formula in clinic, at stepwise doses of 0.1, 1, 3.0, 10.0, 30.0, and 60–100 mL given at 30-minute intervals; the infants were kept in the clinic for 6–8 h to be observed for immediate clinical reactions. For infants without any immediate reactions, oral challenge to CMP-containing formula 200 mL daily was continued for two weeks to observe for any delayed clinical reactions [1,5,6,8,9].

**Table 1:** Basic characteristics, clinical features and laboratory tests in infants with severe FPIES to cow’s milk.

Age (mean ± SD) (Range)	3.2 ± 2.1 Months (29 Days–8 Months)
<6 months	10
6–12 months	2
**Sex, M/F**	9/3
**Feeding patterns**:	
Breast feeding	1
Formula feeding	7
Mixed feeding	4
**Symptoms**	
Diarrhea	12 (100%)
Frequency	5–20 times/day
Consistency	Watery, loose and mucousy
Skin rashes	7 (58.3%)
Erythema	3 (25.0%)
Atopic dermatitis	2 (16.7%)
Angioneurotic edema	2 (16.7%)
Regurgitation/non-bilious vomiting	6 (50.0%)
Projectile vomiting	4 (33.3%)
Fever (T ≥ 38 °C)	6 (50.0%)
Bloating/distention	6 (50.0%)
Blood or mucus in stool	6 (50.0%)
Failure to thrive	5 (41.7%)
Irritability	2 (16.7%)
**Laboratory tests**	
Serum total protein g/L (normal 60–80)	39.9 ± 6.3 (range 25.3–45.5)
Serum albumin g/L (normal 35–50)	21.3 ± 3.5 (range 14.5–24.8)
Serum globulin g/L (normal 20–29)	17.6 ± 4.0 (range 10.8–23.8)
Leukocytosis (≥12.5 × 10^9^/L)	7 (58.3%)
Thrombocytosis (≥440 × 10^9^/L)	7 (58.3%)
Metabolic acidosis	5 (41.7%)
Anemia (HB < 90 g/L)	4 (33.3%)
Peripheral blood eosinophil >6%	4 (33.3%)
Total serum IgE elevation	6 (50.0%)
Milk sIgE (+)	3 (25.0%)
Stool OB (+)	5 (41.7%)
Stool RBC (+)	3 (25.0%)
Stool WBC (+)	5 (41.7%)

Eight of 12 infants had a challenge test after three to six months of CMP avoidance and five patients still had a reaction (two at 100 mL and one each at 1 mL, 30 mL and 90 mL of stepwise doses) including vomiting (1–6 h), skin rash (1–28 h), irritability (0.5 to 3 h) and diarrhea (4 h to 9 days). Nine infants had a challenge test after 9–12 months of CMP avoidance and seven patients were negative. Altogether, 10 infants (83.3%) were tolerant to CMP challenge after 12 months of avoidance and those who were negative for challenge test had no recurrent symptoms when CMP was reintroduced. At clinic follow-up, the remaining two patients who were still positive for CMP challenge eventually became tolerant to CMP challenge at 18 months and 34 months, respectively.

## 4. Discussion

There is an increasing recognition of non-IgE-mediated gastrointestinal food-induced allergic disorders (non-IgE-GI-FAs), which include FPIES, food protein-induced allergic proctocolitis (FPIAP), and food protein-induced enteropathy (FPE) [4]. The most common allergen in infants is cow’s milk. The most prominent clinical features are vomiting, diarrhea, blood and/or mucus stool and dehydration, resulting in hypoalbuminemia and failure to thrive. The diagnosis of FPIES is based on clinical history, sequential symptoms, and timing, after excluding other possible causes [3,4,5,6,7]. Definitive diagnosis requires an oral food challenge test. Unfortunately, the diagnosis of FPIES is frequently delayed or missed because of non-specific symptoms and insufficient definitive diagnostic biomarkers [8]. In addition, FPIES is not well recognized by clinicians; the affected infants are often misdiagnosed as having viral gastroenteritis, food poisoning, sepsis, or a surgical disease. In our study, all of the infants with severe FPIES to cow’s milk presented with diarrhea and hypoalbuminemia. 41.7% of patients had misdiagnosis and delayed diagnosis. An enhanced education for medical practitioners dealing with children about the varied presentations of FPIES is needed [8].

Our study demonstrated that severe FPIES in infants occurred often at <6 months of age with male predominance. All patients responded to CMP avoidance with resolution of symptoms and significant increase of serum albumin, which is consistent with other reports [2,10,11]. The diagnosis of FPIES is most often made on the basis of clinical presentation and CMP avoidance/challenge. Half of our patients had elevated serum total IgE and 25% of them had sIgE to CMP, consistent with a recent large FPIES cohort study by Caubet *et al*., 2014, in which they found that 24% of subjects had detectable specific IgE antibodies to the food that triggered the FPIES and 39% had concomitant IgE sensitization to other foods. They referred to this subgroup of children with detectable specific IgE as “atypical FPIES”, since IgE antibodies to the causal food are typically not detected in subjects with FPIES [3].

The underlying immunological mechanisms for FPIES and hypoproteinemia remain unclear. A consistent finding is shortening of the villi of the jejunum (partial villous atrophy) while consuming milk, which is the basis of the malabsorption. A number of inflammatory changes in intestinal mucosa and increased intestinal permeability result in protein loss [1,4,8,12]. Because tissue biopsy is not routinely required for the diagnosis or management of FPIES, data regarding the gastrointestinal mucosal immune profile in infants with FPIES is often unavailable. All of our 12 patients had hypoalbuminemia and 75% of them had lower serum globulin, suggesting that hypoproteinemia in our patients is likely due to protein-losing enteropathy, rather than a renal disease such as nephrotic syndrome. One limitation of this retrospective study is the lack of fecal α-1 antitrypsin levels due to the unavailability of the test in our institution. FPIES has some clinical features that overlap with other non IgE-mediated gastrointestinal allergic disorders, food protein-induced allergic proctocolitis (FPIAP) and food protein-induced enteropathy (FPE) [4]. Future prospective studies with colonoscopy and histological examination would potentially improve our understanding and diagnosis of those clinical entities.

The strict avoidance of CMP is presently the safest strategy for managing FPIES [1]. In our study, the majority of infants developed a tolerance to CMP challenge after 12 months of avoidance. However, clinical data is still lacking on the length of CMP elimination and the choice of formula, extensively hydrolyzed formula versus elemental formula. Well-controlled randomized clinical trials with sufficient power would be needed to answer those critical questions.

## 5. Conclusions

We report the clinical experience of 12 infants with severe FPIES to cow’s milk. The diagnosis relied on clinical presentation and resolution of symptoms after CMP avoidance. However, FPIES associated with CMPA in infants is not well recognized among pediatricians and misdiagnosis and/or delayed diagnosis is common, resulting in malnutrition, hypoproteinemia, and failure to thrive. Prompt treatment with CMP-free formula is effective and leads to clinical remission of FPIES in infants.
